# Genetic variants of *MICB* and *PLCE1* and associations with the laboratory features of dengue

**DOI:** 10.1186/s12879-017-2514-6

**Published:** 2017-06-09

**Authors:** James Whitehorn, Duong Thi Hue Kien, Nguyen Than Ha Quyen, Bridget Wills, Nguyen Van Vinh Chau, Dong Thi Hoai Tam, Nguyen Minh Tuan, Thomas Jaenisch, Martin Hibberd, Chiea Chuen Khor, Cameron P. Simmons

**Affiliations:** 1 Department of Clinical Research, London School of Hygiene and Tropical Medicine, London, UK; 2 Oxford University Clinical Research Unit, Hospital for Tropical Diseases, 764 Vo Van Kiet, District 5, Ho Chi Minh City, Viet Nam; 3 Centre for Tropical Medicine, Nuffield Department of Medicine, University of Oxford, Oxford, UK; 4 Hospital for Tropical Diseases, 764 Vo Van Kiet, District 5, Ho Chi Minh City, Viet Nam; 5 Children Hospital Number 1, Ho Chi Minh City, Viet Nam; 6 Department of Infectious Diseases, Heidelberg University Hospital, Heidelberg, Germany; 7 Genome Institute of Singapore, Biopolis Way, Singapore, Singapore; 8 School of Public Health, National University of Singapore, Singapore, Singapore; 9 Department of Paediatrics, National University of Singapore, Singapore, Singapore; 10 Department of Ophthalmology, National University of Singapore, Singapore, Singapore; 11 Department of Microbiology and Immunology, University of Melbourne, at the Peter Doherty Institute, Melbourne, VIC 3010 Australia

## Abstract

**Background:**

A previous genome-wide association study identified 2 susceptibility loci for severe dengue at *MICB* rs3132468 and *PLCE1* rs3740360 and further work showed these mutations to be also associated with less severe clinical presentations. The aim of this study was to determine if these specific loci were associated with laboratory features of dengue that correlate with clinical severity with the aim of elucidating the functional basis of these genetic variants.

**Methods:**

This was a case-only analysis of laboratory-confirmed dengue patients obtained from 2 prospective cohort studies and 1 randomised clinical trial in Vietnam (Trial registration: ISRCTN ISRCTN03147572. Registered 24th July 2012). 2742 dengue cases were successfully genotyped at *MICB* rs3132468 and *PLCE1* rs3740360. Laboratory variables were compared between genotypes and stratified by DENV serotype.

**Results:**

The analysis showed no association between *MICB* and *PLCE1* genotype and early viraemia level, platelet nadir, white cell count nadir, or maximum haematocrit in both overall analysis and in analysis stratified by serotype.

**Discussion:**

The lack of an association between genotype and viremia level may reflect the sampling procedures within the included studies. The study findings mean that the functional basis of these mutations remains unclear.

**Trial registration:**

ISRCTN ISRCTN03147572. Registered 24th July 2012.

## Background

Dengue viruses (DENV) cause a spectrum of clinical manifestations ranging from asymptomatic infection through to life-threatening shock and haemorrhage [1, 2]. Single nucleotide polymorphisms (SNPs) at two genomic loci are associated with DSS in Vietnamese children [3]. These SNPs were in the major histocompatibility complex class I polypeptide-related sequence B gene (*MICB*) on chromosome 6 (rs3132468) and in the phospholipase C epsilon 1 (*PLCE 1*) gene on chromosome 10 (rs3740360). Further work demonstrated that these variations are also significantly associated with less severe but medically attended dengue cases dengue [4, 5]. The *MICB* gene encodes a surface protein that contributes to natural killer (NK) cell activation. The observed association with a SNP in the *MICB* gene and dengue shock syndrome (DSS) may reflect dysfunctional NK and CD8 cell activity in severe disease, suggesting a key role for these cells in disease control and pathogenesis. Mutations in *PLCE1* are associated with nephrotic syndrome, a condition characterised by proteinuria, reduced vascular oncotic pressure and subsequent oedema [6]. The association with mutations in *PLCE1* and dengue raises the possibility of a role for this gene in the maintenance of normal endothelial integrity. Despite these speculations, the functional basis for the association of these mutations with DSS remains unclear.

The aim of this study was to measure if an association existed between the aforementioned *MICB* and *PLCE1* specific mutations and virological and hematological features of dengue that have been shown to have correlation with the clinical outcome of infection. For example, the level of viremia in the first days of illness is positively associated with the severity of the disease phenotype [7]. Similarly, the degrees of lymphopenia and thrombocytopenia are associated with development of DSS [8, 9]. Thus this study tested the hypothesis that the *MICB* (rs3132468) and *PLCE1* (rs3740360) variants were associated with clinically important laboratory features of dengue.

## Methods

The specific aims of this analysis were to measure the association between individuals with different *MICB* rs3132468 and *PLCE1* rs3740360 genotypes (wild-type, heterozygous carriers, and homozygous variant) and various laboratory features. The laboratory features that were compared between the groups were (1) early plasma viremia level, (2) platelet nadir, (3) white cell count nadir, and (4) maximum haematocrit.

Samples were obtained from participants who gave written informed consent to participate in the previously reported prospective studies detailed in Table 1 [10–12]. Parents or guardians of the children involved in the studies gave written informed consent on their behalf. All these studies took place in southern Vietnam. The protocols of these studies were reviewed and approved by the Institutional Review Boards of each study site (Hospital for Tropical Diseases Ho Chi Minh City (HCMC), Children’s Hospital 1 and 2 HCMC, and Tien Giang Hospital) and by the Oxford University Tropical Research Ethics Committee. The inclusion criteria for 13DX were (1) fever or history of fever for less than 72 h, (2) clinical suspicion of dengue, (3) 1–15 years of age, (4) written informed consent, and (5) accompanying family member has a mobile phone. The exclusion criteria were (1) deemed unlikely to attend follow-up, and (2) in whom an alternative diagnosis was thought more likely. The inclusion criteria for 22DX were (1) fever or history of fever for less than 72 h, (2) clinical suspicion of dengue, (3) aged 5 years or over, (4) written informed consent. The exclusion criteria were (1) localising features suggesting an alternative diagnosis, and (2) inability to attend daily follow-up. The inclusion criteria for 26DX were (1) age 18 or over, (2) presenting within 72 h of fever onset with an illness consistent with dengue, (3) positive rapid test for dengue non-structural protein 1 (NS1 (NS1 Ag-STRIP, Bio-Rad). The exclusion criteria were (1) alanine transaminase (ALT) level > 150 U/L, (2) creatine kinase (CK) level > 1000 U/L, (3) platelet count <50 × 10^9^/L, (4) pregnancy or lactation, and (5) a history of cirrhosis or myopathy.

**Table 1:** Details of studies included in analysis

Study	Setting	Study type	Age criteria (years)	Number (% male)	Laboratory sampling procedure	Median age (range 5^th^–95^th^ centile)
13DX	Outpatient	Prospective cohort	1–15	1951 (57)	Enrolment only	9 (3–14)
22DX	Outpatient	Prospective cohort	5 or over	749 (60)	Multiple time points	18 (6–39)
26DX	Inpatient	Randomised clinical trial	18 or over	42 (57)	Multiple time points	24 (18–51)

This was a case-only analysis of laboratory-confirmed dengue patients obtained from two prospective cohort studies and one randomised clinical trial in Vietnam. The cohort studies were conducted in both adults and children and aimed to create diagnostic and prognostic algorithms to improve the clinical management of dengue in this setting. The randomised clinical trial was an investigation of lovastatin therapy in adult patients with dengue (Trial registration: ISRCTN03147572) [10].

DNA extractions were performed using a MagNA Pure 96 DNA and Viral NA Small Volume Kit (Roche, Germany) according to the manufacturer’s instructions. Candidate SNPs were genotyped using a TaqMan genotyping assay to amplify and detect the specific alleles in the DNA samples as per the manufacturer instructions. Samples failing picogreen QC (<5 ng/uL in concentration) were excluded from further analysis. Similarly, during the genotyping process, samples, which fail to show adequate allelic discrimination on the Taqman RT-PCR melt-curve, were also excluded from analysis. Each SNP has been shown previously to show good call rate exceeding >95%.

Statistical analysis was performed in Stata 13 (Stata Statistical Software: Release 13. College Station, TX. StataCorp LP). Laboratory variables were compared between genotypes and stratified by DENV serotype using the binary chi-squared test, the Kruskal-Wallis non-parametric test and the analysis of variance test. A sample size calculation was not performed, as a priori*,* the variance of the viremia measurements was not known.

## Results

Two thousand seven hundred forty two dengue cases were successfully genotyped at *MICB* rs3132468 and *PLCE1* rs3740360. 573 cases were in adults (age ≥ 15) and 2180 were in children. There were 109 cases of severe dengue across the cohorts (6 in adults and 103 in children). The baseline characteristics of the study participants are summarised in Table 2.

**Table 2:** Collated study population characteristics (*n* = 2730)

	DENV1 (*n* = 1042)	DENV2 (*n* = 593)	DENV3 (*n* = 263)	DENV4 (*n* = 832)	All (*n* = 2730)
Age (median, IQR), years	10(7–13)	10(7–14)	9(6–13)	11(8–15)	10(7–14)
Sex (n,%)					
Male	596(57.2)	357(60.2)	147(55.9)	474(57.0)	1574(57.7)
Female	446(42.8)	236(39.8)	116(44.1)	358(43.0)	1156(42.3)
Days of illness at enrolment (n,%)					
0	9(0.86)	1(0.17)	0(0)	3(0.36)	13(0.48)
1	323(31.0)	174(29.3)	85(32.3)	195(23.4)	777(28.5)
2	437(41.9)	271(45.7)	113(43.0)	354(42.5)	1175(43.0)
3	269(25.8)	146(24.6)	64(24.3)	279(33.5)	758(27.8)
4	2(0.19)	1(0.17)	1(0.4)	0(0)	4(0.15)
Viremia (mean, 95% CI), log10 copies/mL	7.39(7.29–7.50)	6.89(6.77–7.01)	7.14(6.94–7.33)	6.80(6.71–6.88)	7.08(7.02–7.14)
Clinical classification (n,%)					
Dengue	950(91.2)	553(93.3)	257(97.7)	780(93.8)	2540(93.0)
Severe dengue	39(3.74)	32(5.4)	6(2.3)	32(3.85)	109(3.99)

### Association between MICB rs3132468 and enrolment viremia

The mean viremia was compared between patients with the spectrum of *MICB* genotypes. The mean viremia in patients carrying the C/C allele was 7.16 (95% CI: 6.84–7.47) log_10_-copies/mL. In those with the C/T allele it was 7.05 (95% CI: 6.92–7.18) log_10_-copies/mL, and in those with the T/T allele it was 7.08 (95% CI: 7.01–7.15) log_10_-copies/mL. The difference between these values was not statistically significant. The comparison in viremia levels between *MICB* genotypes was repeated with data stratified by DENV serotype. Again, no significant differences were demonstrated. These data are summarised in Table 3. Collectively, these data indicate some variation in the viremia between the serotypes but no significant difference between the *MICB* genotypes.

**Table 3:** Viremia by *MICB* and *PLCE* genotype by dengue serotype and overall (mean (IQR) (N))

*MICB*				
Serotype	C/C	C/T	T/T	*P*-value
DENV1	7.32 (6.7–7.95) (*n* = 24)	7.28 (7.03–7.53) (*n* = 211)	7.43 (7.31–7.55) (*n* = 761)	0.238
DENV2	7.04 (6.32–7.76) (*n* = 18)	7.03 (6.77–7.29) (*n* = 125)	6.84 (6.7–6.98) (*n* = 450)	0.833
DENV3	6.99 (5.24–8.75) (*n* = 7)	7.15 (6.7–7.6) (*n* = 50)	7.14 (6.91–7.36) (*n* = 206)	0.836
DENV4	7.13 (6.67–7.59) (*n* = 28)	6.75 (6.56–6.93) (*n* = 184)	6.8 (6.7–6.9) (*n* = 620)	0.86
Overall	7.16 (6.84–7.47) (*n* = 78)	7.05 (6.92–7.18) (*n* = 583)	7.08 (7.01–7.15) (*n* = 2081)	0.315
*PLCE*				
Serotype	A/A	A/C	C/C	*P*-value
DENV1	7.38 (7.24–7.53) (*n* = 578)	7.39 (7.22–7.56) (*n* = 397)	7.47 (7.03–7.91) (*n* = 67)	0.972
DENV2	6.83 (6.67–7) (*n* = 334)	6.99 (6.79–7.19) (*n* = 215)	6.83 (6.38–7.29) (*n* = 44)	0.938
DENV3	7.08 (6.82–7.34) (*n* = 157)	7.19 (6.87–7.5) (*n* = 87)	7.32 (6.35–8.29) (*n* = 19)	0.198
DENV4	6.87 (6.75–6.98) (*n* = 472)	6.74 (6.99–7.19) (*n* = 306)	6.51 (6.18–6.83) (*n* = 54)	0.867
Overall	7.08 (7–7.15) (*n* = 1546)	7.09 (6.99–7.19) (*n* = 1012)	7.02 (6.78–7.26) (*n* = 184)	0.7

### Association between PLCE1 rs3740360 and enrolment viraemia

The mean viremia was compared between the *PLCE1* genotypes. The mean viremia in patients carrying the A/A allele was 7.08 (95% CI: 7.00–7.15) log_10_-copies/mL. In those carrying the A/C allele it was 7.09 (95% CI: 6.99–7.19) log_10_-copies/mL, and in those with the C/C allele it was 7.02 (95% CI: 6.78–7.26) log_10_-copies/mL. The difference between these values was not statistically significant. The comparison in viremia levels between *PLCE1* genotypes was repeated with data stratified by DENV serotype. Again, no significant differences were demonstrated. These data are summarised in Table 3. As above, these data indicate some variation in the viremia level between the serotypes but no significant difference between the *PLCE1* genotypes.

### Association between MICB rs3132468 and other laboratory variables

After excluding data from 13DX, which only included enrolment results, the platelet nadir, the maximum haematocrit and the minimum white cell count were compared between the *MICB* genotypes in both an overall analysis and then stratified by serotype. The median platelet nadir in patients with the C/C allele was 82.5 (IQR: 47–101) × 10^9^/L, in those with the C/T allele was 80 (IQR: 48–115) × 10^9^/L, and in those with the T/T allele was 70 (41–113) × 10^9^/L. These differences were not statistically significant. The minimum white cell count in those with the C/C allele was 2.6 (IQR: 2.3–3.1) × 10^9^/L, in those with the C/T allele was 2.9 (IQR: 2.3–3.8) × 10^9^/L and in those with the T/T allele was 2.7 (IQR: 2.1–3.4) × 10^9^/L. These differences were not statistically significant. The median maximum haematocrit in patients with the C/C allele was 45.4% (IQR: 44–49.2%), in those with the C/T allele was 45% (IQR: 42–48.3%), and in those with the T/T allele was 44.7% (IQR: 41.8–48.4%). These differences were not statistically significant. When these data were stratified by DENV serotype again no significant differences were demonstrated. These data suggest no measurable association between *MICB* genotype and the laboratory variables explored in this analysis.

These data are summarised in Table 4.

**Table 4:** Laboratory features by *MICB* and *PLCE* genotype

*MICB*				
Laboratory/clinical feature	C/C (*n* = 18)	C/T (*n* = 150)	T/T (*n* = 554)	*P*-value
Platelet nadir (median(IQR))	82.5 (47–101)	80 (48–115)	70 (41–113)	0.494
Minimum white cell count (median(IQR))	2.6 (2.3–3.1)	2.9 (2.3–3.8)	2.7 (2.1–3.4)	0.214
Maximum haematocrit (median(IQR))	45.4 (44–49.2)	45 (42–48.3)	44.7 (41.8–48.4)	0.244
*PLCE*				
Laboratory/clinical feature	A/A (*n* = 409)	A/C (*n* = 266)	C/C (*n* = 47)	*P*-value
Platelet nadir (median(IQR))	71 (41–109)	76.5 (45–118)	66 (37–114)	0.675
Minimum white cell count (median(IQR))	2.7 (2.1–3.4)	2.8 (2.2–3.6)	2.9 (2.2–3.6)	0.268
Maximum haematocrit (median(IQR))	44.6 (41.9–48.1)	45.1 (42–48.3)	45 (42.4–50.3)	0.336

### Association between PLCE1 rs3740360 and other laboratory variables

The platelet nadir, the maximum haematocrit and the minimum white cell count were compared between the *PLCE1* genotypes in both an overall analysis and then stratified by serotype. The median platelet nadir in patients with the A/A allele was 71 (IQR: 41–109) × 10^9^/L, in those with the A/C allele was 76.5 (IQR: 45–118) × 10^9^/L, and in those with the C/C allele was 66 (37–114) × 10^9^/L. These differences were not statistically significant. The minimum white cell count in those with the A/A allele was 2.7 (IQR: 2.1–3.4) × 10^9^/L, in those with the A/C allele was 2.8 (IQR: 2.2–3.6) × 10^9^/L and in those with the C/C allele was 2.9 (IQR: 2.2–3.6) × 10^9^/L. These differences were not statistically significant. The median maximum haematocrit in patients with the A/A allele was 44.6% (IQR: 41.9–48.1%), in those with the A/C allele was 45.1% (IQR: 42–48.3%), and in those with the C/C allele was 45% (IQR: 42.4–50.3%). These differences were not statistically significant. When these data were stratified by DENV, serotype again no significant differences were demonstrated. These data suggest no measurable association between *PLCE1* genotype and the laboratory variables explored in this analysis.

These data are summarised in Table 4.

## Discussion

This study did not find an association between *MICB* and *PLCE* genotype and early viremia level, platelet nadir, white cell count nadir or maximum haematocrit in both overall analysis and in analysis stratified by serotype. Given the possibility that *MICB* may have an important role in the establishment of antiviral effector function we proposed that mutations in this gene might be associated with higher dengue viraemia. Our analysis in 2742 adults and children with dengue showed no difference in the enrolment viraemia levels between the different *MICB* and *PLCE1* genotypes in both an overall analysis and analysis stratified by DENV serotype. The enrolment viremia was taken in the vast majority of patients in the first 72 h of clinical illness (Table 2). While our understanding of DENV kinetics suggests that this is likely to correspond near to the peak viraemia level it only gives a snapshot of the in vivo viral dynamics. It is possible that the viral clearance patterns varied across genotypes. In addition, measuring plasma viremia only provides a surrogate of what is happening in other tissues – obviously, however, invasive assessments of this would not be appropriate in large prospective studies. However, an interesting finding was the range of early viraemia levels across the DENV serotypes and between the different *MICB* and *PLCE* genotypes. While the observed variation was not statistically significant, it does suggest that there are factors that influence the early viremia level. This may be a combination of intrinsic genetic susceptibility, host immune status and various viral factors [13]. Determining the factors that influence the DENV “set point” would be intriguing and would advance our understanding of dengue pathogenesis.

As no associations between viremia and genotype were shown, it is perhaps not surprising that no associations were demonstrated between genotype and the pre-selected routine haematological laboratory variables both in overall analysis and in analysis stratified by DENV serotype. These variables were chosen because they have been previously shown to have some correlation with dengue severity [8, 9]. However, differences in the study designs, particularly the frequency of laboratory investigations, across the cohorts described meant that the largest study in terms of patient numbers (13DX) was excluded from this component of the analysis.

While previous work has demonstrated an association between genetic variants of *MICB* and *PLCE1* and both severe and non-severe dengue, the functional basis of these associations is not clear [3, 4]. *MICB* encodes an activating ligand for natural killer cells, and possibly CD8+ T cells, raising the possibility that mutations in this gene may result in altered antiviral effector functions and an associated increased viral burden, a recognised risk factor for the development of severe dengue [7]. It is possible that the associations reflect an aspect of dengue pathogenesis unrelated to control of early viral replication. The association of *PLCE1* and dengue is harder to explain. It is interesting to note that mutations in this gene have been associated with nephrotic syndrome, regulation of blood pressure and oesophageal malignancy [6, 14, 15]. While it is plausible that the relationship between mutations in *PLCE1* and both severe dengue and nephrotic syndrome suggests some role in the maintenance of endothelial integrity, it is more difficult to relate this potential function to oesophageal malignancy. The role of *PLCE1* in control of blood pressure warrants further exploration as it raises the possibility of some overlap in function in dengue given hypotension is seen in severe dengue. While the hypotension in dengue shock is mediated largely by plasma leak, it is possible that variants in *PLCE1* play a contributory role. This relationship with diverse conditions suggests an important, but as yet unclear, role for *PLCE1* in human health and disease.

Our study has limitations. The number of patients with the “risk” alleles for *MICB* and *PLCE1* was relatively small, reflecting the low frequency of these allelic variants. The small number of cases may mean that the analysis lacked the power to detect a true association. This is especially true as the dose effect of a given SNP is likely to be small. Further work with a larger sample size may allow further exploration of a potential dose effect of a homozygous allelic variant on initial viremia. In addition, the study methodology varied between the studies included in the analysis meaning that it was only possible to explore the relationship between genotype and laboratory variable in a smaller group of patients. The functional basis of these observed associations remains unclear but it is possible that pooled data from prospective intervention studies will provide clearer insights. The potential offered by this study approach reflects the more frequent sampling that typically occurs in this type of study. It would be intriguing to explore whether these associations have an effect on cytokine levels – this could be explored within the context of prospective studies. Greater insights into dengue host susceptibility have the potential to expand our knowledge of disease pathogenesis and, in the longer term, assist the development of vaccines and therapeutics.

## Conclusions

This study aimed to explore the functional basis of specific mutations in the *MICB* and *PLCE1* genes previously shown to be associated with severe dengue. We assessed whether these specific loci were associated with laboratory features of dengue that correlate with clinical severity. Our analysis showed no association between *MICB* and *PLCE1* genotype and early viraemia level, platelet nadir, white cell count nadir, or maximum haematocrit in both overall analysis and in analysis stratified by serotype. These results mean that the functional basis of these mutations remains unclear. It is possible that greater insights into dengue susceptibility and pathogenesis will be gained from careful analysis conducted within prospective studies.

## Abbreviations

DENV: Dengue virus
DSS: Dengue shock syndrome
HCMC: Ho Chi Minh City
MICB: Major histocompatibility complex class I polypeptide-related sequence B gene
NK: Natural killer cells
PLCE1: Phospholipase C epsilon 1 gene
SNP: Single nucleotide polymorphism
